# Efficient Production of the Flavoring Agent Zingerone and of both (*R*)- and (*S*)-Zingerols via Green Fungal Biocatalysis. Comparative Antifungal Activities between Enantiomers

**DOI:** 10.3390/ijms151222042

**Published:** 2014-12-01

**Authors:** Laura A. Svetaz, Melina G. Di Liberto, María M. Zanardi, Alejandra G. Suárez, Susana A. Zacchino

**Affiliations:** 1 Pharmacognosy Area, School of Biochemical and Pharmaceutical Sciences, National University of Rosario, Suipacha 531, 2000 Rosario, Argentina; E-Mails: lsvetaz@fbioyf.unr.edu.ar (L.A.S.); melinna85@hotmail.com (M.G.D.L.); 2 Institute of Chemistry Rosario (IQUIR)-CONICET, School of Biochemical and Pharmaceutical Sciences, National University of Rosario, Suipacha 531, 2000 Rosario, Argentina; E-Mails: zanardi@iquir-conicet.gov.ar (M.M.Z.); suarez@iquir-conicet.gov.ar (A.G.S.)

**Keywords:** zingerone, chiral zingerols, filamentous fungi, enantioselective bioconversions, green chemistry, antifungal

## Abstract

Zingerone (**1**) and both chiral forms of zingerol (**2**) were obtained from dehydrozingerone (**3**) by biotransformation with filamentous fungi. The bioconversion of **3** with *A. fumigatus*,* G. candidum* or* R. oryzae* allowed the production of **1** as the sole product at 8 h and in 81%–90% at 72 h. In turn, *A. flavus*,* A. niger*,* C. echinulata*,* M. circinelloides* and* P. citrinum* produced **1** at 8 h, but at 72 h alcohol **2** was obtained as the major product (74%–99%). Among them, *A. niger* and *M. circinelloides* led to the anti-Prelog zingerol (*R*)-**2** in only one step with high conversion rates and *ee*. Instead, *C. echinulata* and *P. citrinum* allowed to obtain (*S*)-**2** in only one step, with high conversion rates and *ee.* Both chiral forms of **2** were tested for antifungal properties against a panel of clinically important fungi, showing that (*R*)-, but not (*S*)-**2** possessed antifungal activity.

## 1. Introduction

Nearly 80% of all available flavoring agents used worldwide are produced by chemical synthesis [1]. However there is an increasing preference of the consumer for “natural” food additives all over the world, mainly due to increasing health and nutrition conscious lifestyles [2]. This trend is reflected in European and US legislations [3] that clearly discriminate between natural or synthetic (natural-identical) flavors, turning the interest of industries to the production of natural flavors. In addition, these legislations consider that the strategy of production of flavors by microbial processes (namely white biotechnology or green chemistry) can be labeled as “natural” [4].

Zingerone [4-(3-methoxy-4-hydroxyphenyl)-butan-2-one] CAS#122-48-5 (**1**) is the least pungent component of *Zingiber officinale* Rosc. [5,6] that provides the typical ginger sweet spicy aroma [7] and is used as a flavoring agent in the food industry. Simultaneously it also minimizes the lipid oxidation of food, which strongly suggests that **1** can be used as an antioxidant and food preservative [8]. This phenolic alkanone is also present in wines at very low concentrations (0.74–8.94 × 10^−3^ µg/mL) [9]. In addition to the applications as a food additive, **1** possesses several pharmacological effects, such as antioxidant, anti-inflammatory, anticancer, antiemetic, anxiolytic, antithrombotic, radiation protective, antimicrobial, immunostimulant enhancer and appetite stimulant [7,10,11], also inhibiting the formation of reactive nitrogen species which are important factors for Alzheimer’s disease [12]. An overview on patents related to ginger and its components [13] pointed out the potential value of ginger products including **1**, for pharmaceutical applications.

In turn, zingerol [4-(4-hydroxy-3-methoxyphenyl)-butan-2-ol] CAS#39728-80-8 (**2**) is a natural non-pungent alcohol [14], isolated as levorrotatory 2*R*-aglycone from the needles of *Taxus baccata* L. [15] (yield 0.001%) and it is also naturally produced for female sexual attraction (in a non-reported stereochemical form) by *Bactrocera dorsalis* male fly upon consumption of **1** from *Bulbophyllum* flowers [16]. Regarding its heterosides, dextrorotatory 4-*O*-glucoside was isolated from *Oxytropis myriophylla* [17] in which the C-2 absolute configuration is not reported and, recently, 4-*O*-glucoside with 2*S*-aglycone, namely bumaldoside C, was isolated from *Staphylea bumalda* [18]*. Rac*-**2** demonstrated to be an inhibitor of colonic motility in rats, being suggested as an oral or suppository medicine for treating diarrhea and other intestinal disorders [14]. It has been also claimed as a component of mixtures containing caffeic, chlorogenic or ferulic acids for preventing, improving or treating hypertension [19]. In addition, **2** (of unspecified C-2 absolute configuration) was disclosed in different patents as an oral mucosa stimulating component of a formula of a throat care agent [20,21]. *Rac*-**2** has also demonstrated to be a useful substrate for the stereoselective spiroannulation to asymmetric oxaspiro compounds (diastereoisomeric ratios not over 65/35), that are useful starting materials for the asymmetric synthesis of manumycin-type natural products [22].

Ketone **1** was previously obtained from dehydrozingerone [(*E*)-4-(4-hydroxy-3-methoxyphenyl) but-3-en-2-one] CAS#1080-12-2 (**3**), by catalytic hydrogenation of the double bond with H_2_/Raney Ni [23] or H_2_/Pd-C [24].

In turn, previous attempts to synthesize chiral (*S*)- and (*R*)-**2** led only to the (*S*)-enantiomer in low yields and variable *ee*, suggesting that the hydrogen was transferred by the ketone *re-*face, following the Prelog’s rule [25]. So, (*S*)-(+)-**2** was previously obtained as a minor by-product (15%, 98% *ee*) in the asymmetric reduction of **1** to 4'-*O-* or 2-*O*-glucosides with cultured cells of *Phytolacca americana* [26] and by biotransformation of **1** with baker’s yeast (45%, 65% *ee*) [15]. In contrast, a route to (*R*)-**2** has not been described yet, except in the work of Kitayama *et al*. [27] who claimed that (*R*)-**2** was obtained along with (*S*)-**2** by kinetic resolution of *rac*-**2** through lipase-catalyzed transesterifications. However, those transesterifications did not lead to free (*R*)-**2** but to its acetate. Then, free chiral (*R*)-**2** was obtained by hydrolysis of the acetate, but the whole reaction involved three steps: (i) reduction of **1** with sodium borohydride to give *rac*-**2**; (ii) lipase-transesterification of *rac*-**2** to its chiral (*R*)-acetate; (iii) hydrolysis of the (*R*)-acetate to give free (*R*)-**2** (6%–53% overall yields and 70%–99% *ee*).

In this paper we present a biocatalytic route to obtain “natural” **1** and both (*R*)- and (*S*)-**2** in only one step from **3** through biotransformation with filamentous fungi. The predominant driving forces for selecting this fungal panel were that some of these fungi have previously given rise to bio-reduction products that have the opposite stereochemistry to the predicted by Prelog [28], or have demonstrated enoate-reductase ability in our previous works [29].

It is worth taking into account that the production of (*R*)- and (*S*)-**2** could constitute a great advance in the pharmaceutical as well as in the synthetic fields since all applications reported for **2** were referred to *rac*-**2**, that is easily obtained by borohydride reduction of **1** [30]. Since the biological activity differences between enantiomers are well-known [31,32], a simple, inexpensive and clean mode of production of both forms of chiral **2** will be of great importance to differentiate their biological activities. In order to provide a proof of the behavior differences of both enantiomers, here we present the antifungal activity of (*R*)- and (*S*)-**2** against a panel of clinically important fungi with standardized methodologies.

## 2. Results

### 2.1. Fungal Biotransformation of **3**

**Scheme 1:**
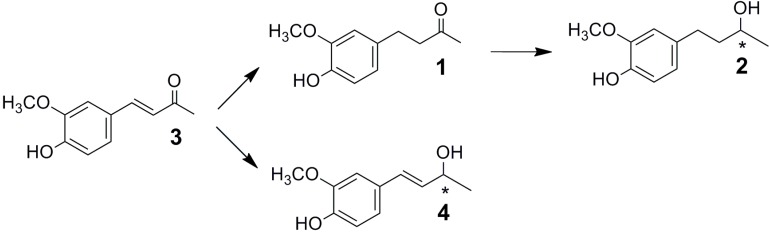
Possible products in the biotransformation of dehydrozingerone **3**. * Chiral carbon.

Dehydrozingerone (**3**), a natural α,β-unsaturated ketone previously isolated from *Z. officinale* [33], was submitted to fungal biotransformation with the panel of filamentous fungi listed in Table 1, by using growing cells methodology [34]. The possible biotransformation products of **3** depend on the activity of two enzymes, enoate-reductase (ER) that would lead to the saturated ketone **1** and alcohol dehydrogenase (ADH) that would reduce the carbonyl group leading to the saturated alcohol **2** or the allylic alcohol (**4**) (Scheme 1).

### 2.2. Biotransformation of **3** with the Whole Fungal Panel

The GC-MS analysis of the biotransformation crude products showed that, after 72 h (Table 1), Aspergillus flavus, Aspergillus fumigatus, Aspergillus niger, Cunninghamella echinulata, Geotrichum candidum, Mucor circinelloides, Penicillium citrinum, Rhizopus oryzae and Trichosporum cutaneum produced an almost complete substrate consumption (98%–99% conversion rates) while Fusarium graminearum got a modest 51% (Table 1). The two new-formed compounds have mass fragmentations with molecular peaks of [M]^+^ 194 and 196, respectively, and proved to be ketone **1** (Retention Time (RT) = 6.95 min in GC-MS) and the saturated alcohol **2** (RT = 7.09 min). No allylic alcohol **4** (RT = 7.47 min) was generated in the biotransformations (Scheme 1). To know the RT of **4**, we synthesized this allylic alcohol as reported in Materials and Methods and submitted it to GC-MS in the same conditions as the rest of compounds and the biotransformation products. The non-appearance of allylic alcohol was also observed by Fronza *et al*. in the biotransformation of the closely related structure 4-(4-hydroxyphenyl)-but-3-en-2-one with baker’s yeast [35].

**Table 1:** Biotransformation of dehydrozingerone **3**. Products distribution (in %) of zingerone **1** and chiral zingerol **2** after fed with **3** at 0.5 mg/mL with filamentous fungi. Absolute configuration, optical rotations (α) and enantiomeric excess of **2** are included.

Entries	Fungal spp.	Conversion Rate (%)	1 (%)	2 (%)	Absolute Configuration	${\left[\alpha \right]}_{D}^{25}$ (0.75, CHCl_3_)	*ee* (%)
1	*A. flavus*	>99	26	74	*S*	+3.1 ± 0.2	20
2	*A. fumigatus*	>99	90	10	*S*	+2.3 ± 0.4	14
3	*A. niger*	99	1	98	*R*	−15.5 ± 0.7	94
4	*C. echinulata*	>99	1	99	*S*	+11.3 ± 0.7	70
5	*F. graminearum*	51	50	1	*R*	−11.8 ± 0.4	72
6	*G. candidum*	98	84	14	*R*	−16.1 ± 0.5	98
7	*M. circinelloides*	>99	1	99	*R*	−10.4 ± 0.7	64
8	*P. citrinum.*	>99	2	98	*S*	+11.7 ± 0.8	94
9	*R. oryzae*	>99	81	19	*S*	+14.2 ± 0.4	88
10	*T. cutaneum*	>99	57	43	*R*	−4.6 ± 0.3	28

*A. flavus*: *Aspergillus flavus* ATCC 9170; *A. fumigatus*: *Aspergillus fumigatus* ATCC 26934; *A. niger*: *Aspergillus niger* ATCC 9029; *C. echinulata*: *Cunninghamella echinulata* CCC 164; *F. graminearum*: *Fusarium graminearum* CCC 170; *G. candidum*: *Geotrichum candidum* CCC 116; *M circinelloides*: *Mucor circinelloides* CCC 128; *P. citrinum*: *Penicillium citrinum* CCC 129; *R. oryzae*: *Rhizopus oryzae* CCC 130; *T. cutaneum*: *Trichosporum cutaneum* CCC 133.

Table 1 shows the products distribution at 72 h (% of **1** and **2**) after each biotransformation procedure (columns 4 and 5). In this Table the absolute configuration, optical rotations (α) and enantiomeric excess of **2** (discussed in Section 2.4) were included.

**Figure 1:**
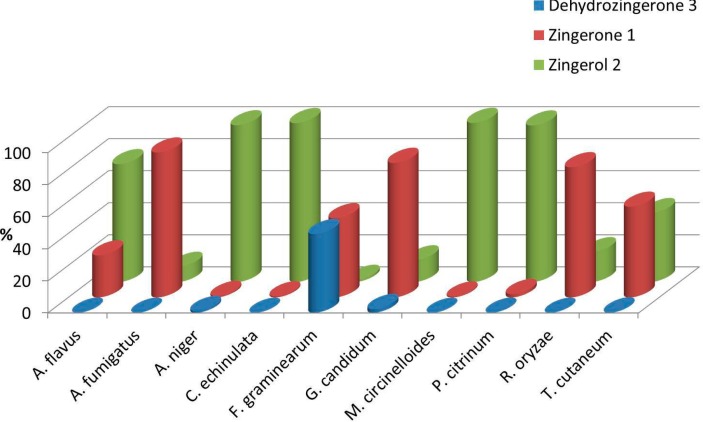
Biotransformation of dehydrozingerone **3** with ten different filamentous fungi during 72 h. The percentages (%) of each product zingerone **1** or zingerol **2** are showed in different rows. The front row shows the percentage of the remainder substrate **3** in each biotransformation process*.*

*A. fumigatus*,* G. candidum* and* R. oryzae* selectively produced **1** in 81%–90% at 72 h while *A. flavus*,* A. niger*,* C. echinulata*,* M. circinelloides* and* P. citrinum* selectively produced **2** in 74%–99% at 72 h. *T. cutaneum* was the only fungus that produced **1** and **2** in similar amounts (57% and 43%, respectively). For the sake of clarity, Figure 1 shows the results of the biotransformation of **3** at 72 h with different filamentous fungi in a bar graph.

### 2.3. Time-Course of the Biotransformation of **3** with Selected Fungi

**Figure 2:**
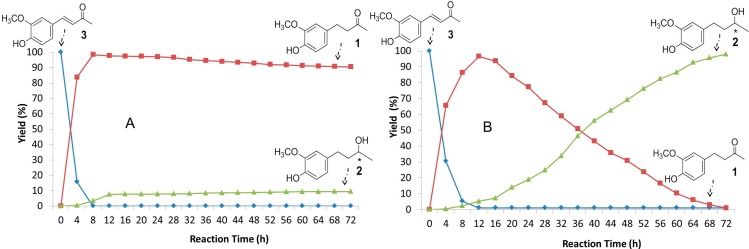
Time-course of the biotransformation of dehydrozingerone **3** with *A. fumigatus* (**A**) and *A. niger* (**B**). * Chiral carbon.

The course of the reduction of **3** was followed during 72 h, for two selected fungi representative each one of those that produced predominantly **1** (*A. fumigatus*) or **2** (*A. niger*). An aliquot of 10 mL was extracted every 4 h from the culture with EtOAc and the extract was analyzed by GC-MS (Figure 2).

It is clear that the time-courses of the reactions with both fungi have the common feature that, at about 8 h, **3** was almost completely consumed, and **1** was produced as the result of ER activity.

However, each time-course continues in a quite different way after 8 h. At 72 h, saturated ketone **1** showed to be the major biotransformation product (90%) with *A. fumigatus* (Figure 2A) being **2** produced in only 10%, evidencing a very low ADH activity for this fungus. In contrast, *A. niger* gradually reduced, after 8 h, the whole amount of ketone **1** to **2** (Figure 2B) being **2** at 72 h almost the only product (98%), thus indicating that both enzymes ER + ADH are present in this fungus and they act one after other.

### 2.4. Absolute Configuration of the Obtained Alcohols **2**

**Figure 3:**
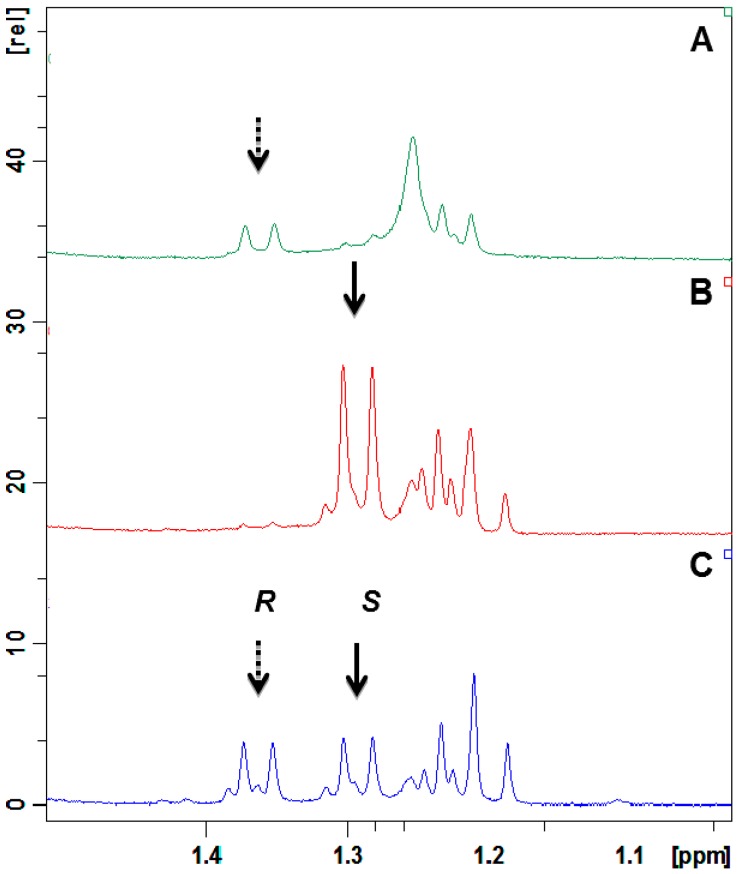
^1^H NMR spectra of (*S*)-(−)-MTPA esters with: (**A**) a mixture enriched with the alcohol (*R*)-**2** obtained by biotransformation of **3** with *A. niger*; (**B**) a mixture enriched with the alcohol (*S*)-**2** obtained by biotransformation of **3** with *P. citrinum*; and (**C**) *rac*-**2**.

**Figure 4:**
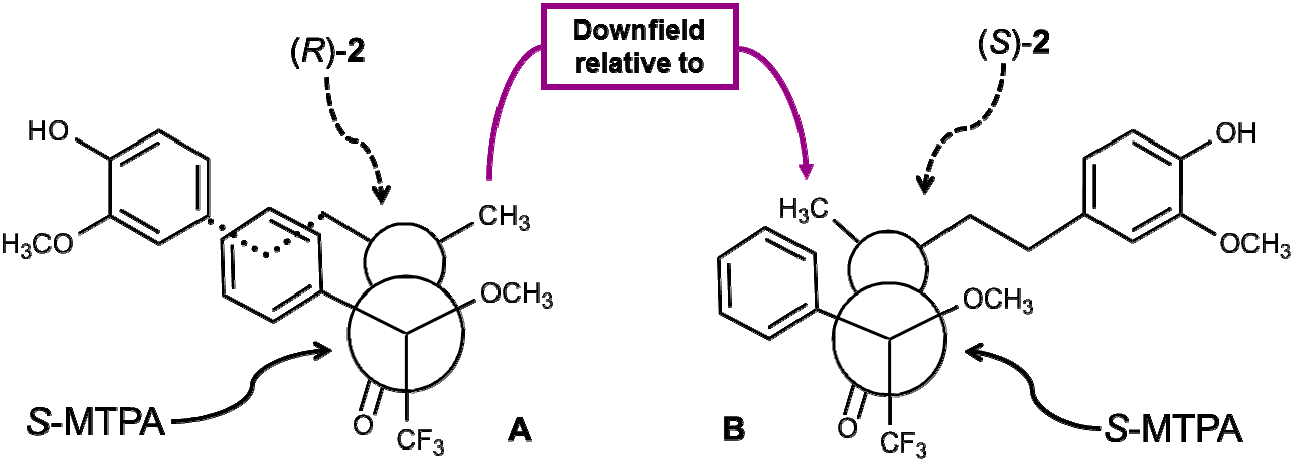
Configuration correlation model for (*S*)-MTPA esters of (*R*)-**2** (**A**) and (*S*)-**2** (**B**).

Although the absolute configuration and the *ee* could be calculated from the optical rotations of (*R*)-**2** reported in the literature [15,27], both previous papers differ in the values of optical rotations. So, we decided to corroborate the absolute configuration and determine the *ee* of our biotransformation products by preparing *rac*-**2** by NaBH_4 _reduction of **1** and observing the ^1^H NMR spectral differences of the diastereomeric esters formed with the (*S*)-α-methoxy-α-trifluoromethylphenyl acetic acid (MTPA) and each (*R*)-**2** and (*S*)-**2** enantiomeric alcohols of the racemic mixture. Results showed that methyl protons of (*R*)-**2** esterified with (*S*)-MTPA appear as doublets at lower fields (1.36 ppm) than those of (*S*)-**2** (1.29 ppm) (Figure 3) because the methyl protons of the (*S*)-form are shielded by the phenyl group of (*S*)-MTPA (Figure 4).

**Figure 5:**
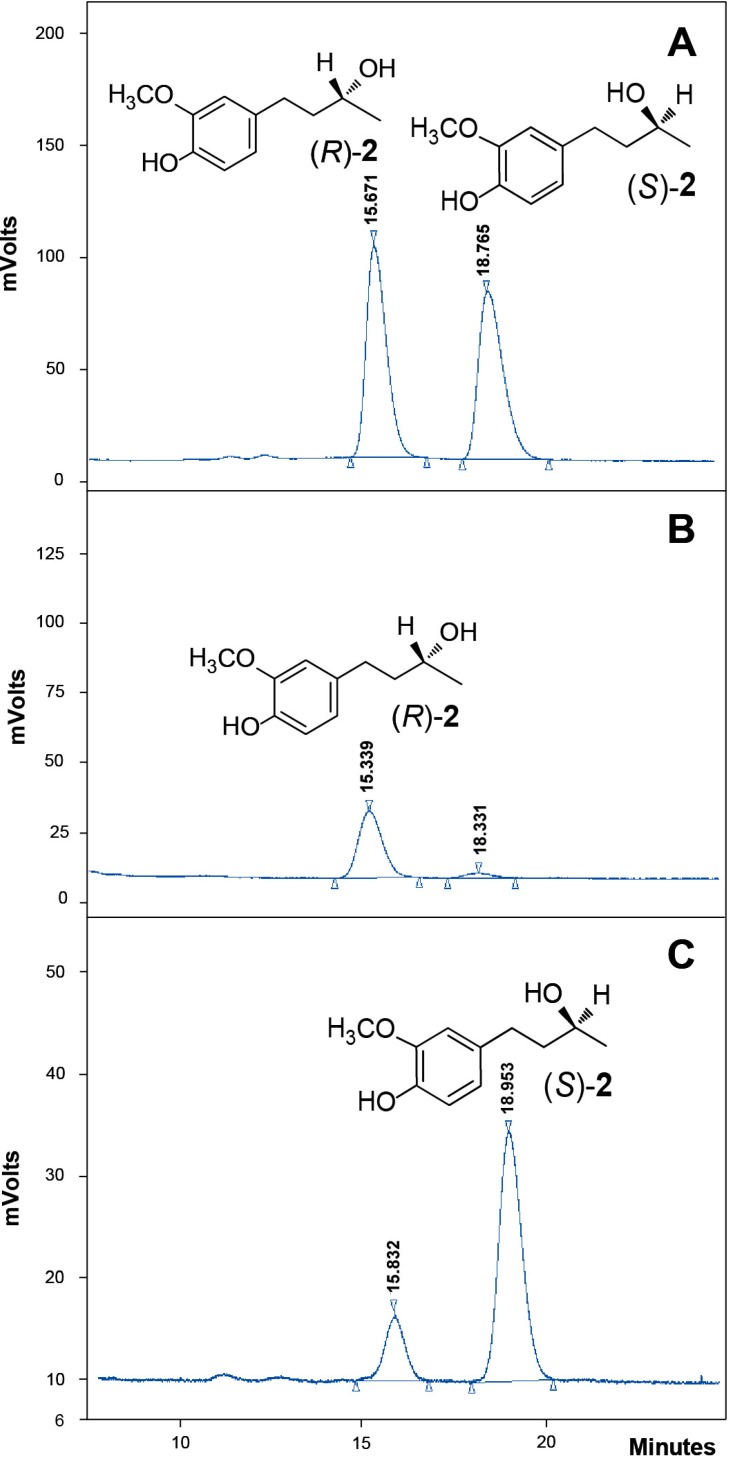
Chromatograms obtained by chiral HPLC of *rac*-**2** (**A**); (*R*)-**2**, Retention Time (RT) = 15.7 min (**B**) and (*S*)-**2**; RT = 18.7 min (**C**).

The enantiomeric excess (% *ee*) of the products was determined with chiral HPLC, by using a Chiralcel OD-H column. Figure 5 shows HPLC profiles of *rac*-**2** (Figure 5A), and of the products enriched in (*R*)- or (*S*)-**2** obtained by biotransformation with *A. niger* (Figure 5B) and *P. citrinum* (Figure 5C). The retention times (RT) for each enantiomer (*R*)- and (*S*)-**2** were 15.7 and 18.7 min, respectively.

Through this methodology, we could corroborate the stereochemistry of all obtained alcohols and determine the *ee* (Table 1). Among the five fungi (*A. flavus*,* A. niger*,* C. echinulata*,* M. circinelloides* and* P. citrinum*) that selectively produce **2**, *A. flavus*, *C. echinulata* and *P. citrinum* produced mainly (*S*)-(+)-**2** with 20%, 70% and 94% *ee*, respectively. Instead, *A. niger* and *M. circinelloides* gave the levorotatory (*R*)-(−)-**2** with 94% and 64% *ee*, respectively.

### 2.5. Antifungal Activity of (S)-, (R)- and rac-**2**

**Figure 6:**
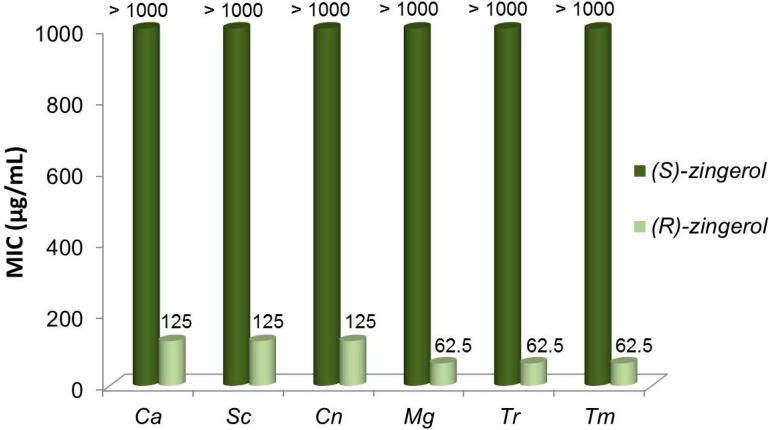
Comparative antifungal activities of (*S*)- (94% *ee*) and (*R*)-zingerol (98% *ee*) against yeasts and filamentous fungi. *Ca*: *Candida albicans* ATCC 10231; *Sc*: *Saccharomyces cerevisiae* ATCC 9763; *Cn*: *Cryptococcus neoformans* ATCC 32264; *Mg*: *Microsporum gypseum* CCC 115; *Tr*: *Trichophyton rubrum* CCC 110; *Tm*: *Trichophyton mentagrophytes* ATCC 9972. MICs of terbinafine against *Mg*, *Tr* and *Tm* = 0.04, 0.01 and 0.04 µg/mL, respectively. MICs of Amphotericin B against *Ca*, *Sc* and *Cn* = 1.00, 0.50 and 0.25 µg/mL, respectively.

Considering that the stereochemistry of chiral compounds often play an important role in the antimicrobial activity [36], we tested (*R*)- and (*S*)-**2** against a panel of clinically important fungi, comprising the yeasts *Candida albicans*, *Saccharomyces cerevisiae* and *Cryptococcus neoformans* and the dermatophytes *Microsporum gypseum*, *Trichophyton rubrum* and *Trichophyton mentagrophytes* (Figure 6). For testing, we chose (*S*)-**2** with 94% *ee* and (*R*)-**2** with 98% *ee* (Table 1). The minimum concentration that inhibited fungal growth (MIC) was investigated following the guidelines of Clinical and Laboratory Standards Institute (CLSI) [37,38].

Results showed that (*S*)-**2** was completely devoid of activity up to 1000 µg/mL. Instead, (*R*)-**2** possessed antifungal activity against *C. albicans*, *S. cerevisiae* and *C. neoformans* with MIC values = 125 µg/mL and against the dermatophytes* M. gypseum*,* T. rubrum* and *T. mentagrophytes* with MICs = 62.5 µg/mL.

## 3. Discussion

In this work, the α,β-unsaturated ketone **3** was used as the substrate of biotransformation to obtain **1** and both chiral forms of **2**, mainly of (*R*)-**2**. Neither the substrate **3** nor filamentous fungi have been previously used to obtain chiral **2**.

The selective reduction of each the carbonyl group or/and the double bond can be achieved in compounds containing two reducible functional groups, such as the α,β-unsaturated ketone **3**, depending whether the biocatalyst possesses ER or ADH enzyme or both [28]. The presence of both enzymes could be a bottleneck in the biotransformations with whole cells, because they can act one after other in two steps in which the product of the first reaction is the substrate for the second activity or, instead, one can compete with the other, sometimes preventing the generation of the desired product or/and the stereoselectivity of the reaction as it was previously reported [35,39,40,41].

Interesting enough, the biotransformation of **3** with *A. fumigatus*,* G. candidum* and* R. oryzae* gave selectively **1** at 72 h with 81%–90% strongly suggesting that these fungi possess a high ER but modest ADH activities. In addition, the time-course of the reaction with *A. niger* suggests that the bioconversion with this fungus and also with *A. flavus*,* C. echinulata*,* M. circinelloides* and* P. citrinum* would allow the production of **1** as the sole product if the reaction is stopped at short times.

In turn, the biotransformation of **3** with *A. flavus*,* A. niger*,* C. echinulata*,* M. circinelloides* and* P. citrinum* produced **2** at 72 h in 74%–99%, showing that these fungi possess both ER and ADH enzymes, although ADH acts after ER. However, different enantioselectivities were obtained with each fungus. *A. niger* and *M. circinelloides* reduced the carbonyl group in the anti-Prelog sense giving (*R*)-(−)-**2**. Of them *A. niger* was the most efficient catalyst in terms of alcohol **2** yield and *ee* (98% yield and 94% *ee*). According to our knowledge, this is the first report on filamentous fungi (or any microorganism) able to produce (*R*)-**2** with good yields and high *ee* in only one step-biotransformation procedure.

Instead, *A. flavus*,* C. echinulata* and *P. citrinum* reduced the carbonyl group in the Prelog sense producing (*S*)-(+)-**2**, being *P. citrinum* the best biocatalyst with alcohol **2** yield = 98% and *ee* = 94%.

The antifungal properties of both chiral forms of **2** against a panel of clinically important fungi showed that (*R*)*-*, but not (*S*)-**2**, possessed antifungal activity, mainly active against dermatophytes. These fungi are responsible for approximately the 80%–93% of chronic, recurrent and hardly eradicable skin infections in human beings and are the ethiological agents of *tinea unguium* (producer of invasive nail infections), *Tinea manuum* (palmar and interdigital areas of the hand infections) and *Tinea pedis* (Athlete’s foot), the last one being the most prevalent fungal infection in developed countries, and the first one accounting for 50% and 90% of all fingernail and toenail infections, respectively [42].

Although the antifungal activity of (*R*)-**2** is moderate, the great difference in activity between both enantiomers of **2** provides a clear proof on the relevance of having achieved an easy and inexpensive route to the production of this alcohol, in high conversion rates and *ee*. In the synthetic field, the spiroannulation of each enantiomer of **2** will allow a more efficient production of asymmetric oxaspiro compounds that are useful starting materials for the asymmetric synthesis of manumycin-type natural products [22].

## 4. Experimental Section

### 4.1. General Considerations

Solvents and reagents were purchased from Sigma (St. Louis, MO, USA) and were purified according to standard procedures. ^1^H and ^13^C NMR spectra were recorded on a Bruker Avance-300 DPX spectrometer (Karlsruhe, Germany). Compounds were dissolved in deuterated solvents from commercial sources (Sigma) using tetramethylsilane (TMS) as internal standard. Chemical shifts are reported in ppm (δ) relative to the solvent peak (CHCl_3_ in CDCl_3_ at 7.26 ppm for protons and at 77.0 ppm for carbons). Signals are designated as follows: *s*, singlet; *d*, doublet; *dd*, doublet of doublets; *m*, multiplet. Gas chromatograms were obtained in a CG-MS Shimadzu QP-2010 plus (Shimadzu, Kyoto, Japan), column SPB-1 30 m × 0.25 mm of inner diameter, film 0.25 μm, ionization energy 70 eV.

Optical rotations were measured in a Jasco DIP-1000 digital polarimeter (Easton, MD, USA). The reported data refer to the Na-line value (589 nm). IR spectra were recorded using an IR Prestige-21 Fourier Transform Infrared Spectrophotometer (Shimadzu, Kyoto, Japan). HPLC analyses were performed with a chromatograph Varian ProStar (San Diego, CA, USA) equipped with UV-V detector ProStar 320 at 254 nm, using a Chiralcel OD-H, 25 cm column. Hexane (Hex) and isopropanol HPLC grade were used as eluents in a mixture 92:8. Flow rate was 0.8 mL/min.

### 4.2. Preparation of **3**

A NaOH aq solution (20%, 50 mL) was added to a solution of vanillin (Sigma) (10.0 g, 65.7 mmol) in acetone (60 mL), and the mixture was stirred overnight [43]. Then, the mixture was diluted with cold distilled water and acidified with cc HCl (70 mL) leading to a yellow precipitate, which was filtered and purified by Silica gel column chromatography by using a mixture of Hex and EtOAc (9:1) as eluent. Pure **3** (10.6 g, 84%) was obtained as a colorless solid, mp 126–127 °C. Spectroscopic data were in agreement with those previously described [43,44,45]. IR ν_max_/cm^−1^ (film) 3352, 1587, 1257. ^1^H NMR (CDCl_3_; 300 MHz): δ 2.37 (3H, *s*, 1-H); 3.93 (3H, *s*, OMe); 5.94 (1H, *s*, 4’-OH); 6.59 (1H, *d*, *J* = 16.2 Hz, H-3); 6.93 (1H, *d*, *J* = 8.1 Hz, H-5’); 7.06 (1H, *d*,* J* = 1.9 Hz, H-2'); 7.09 (1H, *dd*, *J* = 8.1; 1.9 Hz, H-6'); 7.45 (1H, *d*, *J* = 16.2 Hz, H-4). ^13^C NMR (CDCl_3_; 75 MHz): δ 27.3 (1-Me); 56.0 (7'-OMe); 109.3 (C-2'); 114.8 (C-5'); 123.5 (C-6'); 125.0 (C-3); 126.9 (C-1'); 143.7 (C-4); 146.9 (C-4'); 148.3 (C-3'); 198.4 (C=O). GC:RT = 7.74 min. EI-MS *m*/*z* 192 [M^+^].

### 4.3. Fungal Strains Used for Biotransformation

The filamentous fungi* A. flavus* ATCC 9170; *A. fumigatus* ATCC 26934; *A. niger* ATCC 9029; *C. echinulata* CCC 164; *F. graminearum* CCC 170; *G. candidum* CCC 116; *M. circinelloides* CCC 128; *P. citrinum* CCC 129; *R. oryzae* CCC 130 and *T. cutaneum* CCC 133 were obtained from either the American Type Culture Collection (ATCC) (Manassas, VA, USA) or the Culture Collection of Reference Center on Mycology (CCC, School of Biochemical and Pharmaceutical Sciences, Suipacha 531-(2000)-Rosario, Argentina). Fungi were grown on agarized Czapeck (for *Aspergillus* spp*.*) or Sabouraud culture medium (for the rest of fungal strains) for 3 days at 30 °C until well-sporulated. Each fungus inoculum suspension was prepared with conidia taken from the culture surface and adjusted to 2–5 × 10^6^ CFU/mL, according to reported procedures [37].

### 4.4. Biotransformation Methodology

In two-liter erlenmeyer-flasks containing Czapek or Sabouraud broth medium (1 L), fungal inoculum (1 mL) was added and the mixture was incubated at 30 °C for 72 h with shaking on an Innova 4000 orbital shaker New Brunswick Scientific (Edison, NJ, USA) at 150 rpm [34]. A solution of **3** (100 mg) in DMSO (5 mL) was added to one of the flasks (bioconversion flask) and instead, 5 mL of pure DMSO was poured in the other one (fungal growth control flask). A third flask (substrate control flask), containing culture medium and substrate (fungal-free) was prepared. The three flasks were incubated with stirring (150 rpm) at 30 °C for 72 h. After incubation, mixtures were filtered and aqueous phases were bulked and extracted with EtOAc (3 × 250 mL). Organic phases were dried over Na_2_SO_4_.

### 4.5. Analysis of the Product Mixtures with GC-MS

The products were analyzed by GC-MS using a Shimadzu QP-2010 plus chromatograph (Shimadzu, Kyoto, Japan), equipped with a fused silica gel column (SPB-1 30 m × 0.25 mm ID) with He as a carrier gas at a flow rate of 1.0 mL/min, coupled to a mass selective detector, 0.25 µm film, and an ionization energy of 70 eV with a temperature program of 50–300 °C at 25 °C/min; total time 13 min.

The conversion rates were determined by using the following equation: [Conversion % = (product TIC/product TIC + substrate) × 100] (TIC = total ion current). The product mixtures were purified by PTLC with silica gel GF254 (Merck, Buenos Aires, Argentina) by using a mixture of Hex:EtOAc (1:1) as eluent. The structure of each product was elucidated by ^1^H NMR, ^13^C NMR and MS and the spectra were compared with the literature.

### 4.6. Preparation of MTPA Esters of (R)-, (S)- and rac-**2**

Chiral alcohol **2** and *rac*-**2** were converted into MTPA esters with (*S*)-(−)-MTPA [as its (*R*)-(−)-MTPA-Cl] (Sigma) following reported procedures [46]. The reaction mixture was shaken and allowed to stand 12 h at room temperature (rt). Then, 3-dimethylamino-1-propylamine (DMAPA) was added in excess and the mixture was allowed to stand for 5 min before being diluted with diethyl ether. The organic solution was washed with cold 1 N HCl (2 mL), cold saturated Na_2_CO_3_ solution (2 mL) and brine (2 mL), dried (MgSO_4_) and concentrated to dryness. The residue was analyzed by ^1^H NMR (CDCl_3_) [47].

### 4.7. Synthesis of Ketone **1** for Comparative Purposes

To a stirred solution of **3** (5 g) in EtOAc (30 mL), Pd/C (10%) was added and the mixture was stirred 4 h under H_2_ atmosphere (1 atm) at rt. The reaction mixture was filtered, washed with EtOAc and evaporated under vacuum giving a crude product which was purified by column chromatography using Hex:EtOAc (8:2) as eluent to obtain **1** (4.2 g, 83%) as a colorless solid, mp 40–42 °C [48]. Spectroscopic data of **1** were in good agreement with those previously described [49]. IR ν_max_/cm^–1^ (film) 3408, 1708, 1271. ^1^H NMR (CDCl_3_; 300 MHz): δ 2.15 (3H, *s*, H-1); 2.79 (4H, *m*, H-3, H-4); 3.88 (3H, *s*, 3'-OMe); 5.59 (1H, *s*, 4'-OH); 6.69 (2H, *m*, H-2' and H-6'); 6.84 (1H, *d*, *J* = 8.1 Hz, H-5'); ^13^C NMR (CDCl_3_; 75 MHz): δ 29.5 (C-4); 30.1 (CH_3_); 45.5 (C-3); 55.1 (3'-OCH_3_); 111.1 (C-2'); 114.4 (C-5'); 120.7 (C-6'); 132.9 (C-1'); 143.9 (C-4'); 146.4 (C-3'); 208.3 (C=O). GC:RT = 6.95 min. EI-MS *m*/*z* 194 [M^+^].

### 4.8. Synthesis of rac-(±)-**2** for Comparative Purposes

NaBH_4_ (23 mg, 0.6 mmol) was added in small portions to a stirred, cooled (0 °C) solution of **1** (38 mg, 0.2 mmol) in MeOH (3 mL) [15]. The mixture was stirred 3 h at 0 °C and 1 h at rt. Water and a few drops of cc HCl were then added and the mixture was extracted with Et_2_O (3 × 5 mL). The combined Et_2_O extracts were washed with saturated aq NaHCO_3_ and water, dried (Na_2_SO_4_) decanted and evaporated to dryness yielding 35.3 mg (89%) of a solid from which pure (±)-**2** was obtained by preparative TLC [plates with Si gel 60 GF_254_ (Merck, Argentina) using a mixture of hexane (Hex) and EtOAc (7:3) as eluent]. IR ν_max_/cm^−1^ (film), 3340, 1271. ^1^H NMR (CDCl_3_; 300 MHz): δ 1.23 (3H, *d*, *J* = 6.2 Hz, CH_3_); 1.74 (2H, *m*, H-3); 2.63 (2H, *m*, H-4); 3.83 (1H, *m*, H-2); 3.88 (3H,* s*, 3'-OMe); 6.72 (1H, *dd*,* J* = 8.0 and 1.8 Hz, H-6'); 6.78 (1H, *d*,* J* = 1.8 Hz, H-2'); 7.05 (1H, *d*, *J* = 8.0 Hz, H-5'); ^13^C NMR (CDCl_3_; 75 MHz): δ 20.2 (CH_3_); 31.2 (C-3); 38.6 (C-4); 55.8 (3'-OCH_3_); 68.7 (C-2); 110.0 (C-2'); 114.3 (C-5'); 120.9 (C-6'); 133.7 (C-1'); 143.7 (C-4'); 146.4 (C-3'). GC:RT = 7.09 min. EI-MS *m*/*z* 196 [M^+^].

### 4.9. Synthesis of Allylic Alcohol **4** for Comparative Purposes

The α-enone **3** (168 mg, 0.87 mmol) and CeCl_3_·7H_2_O (326 mg, 0.87 mmol) were dissolved in 4 mL of MeOH. NaBH_4_ (29 mg, 0.75 mmol) was added in one portion, with stirring (30 min) [50]. Stirring was continued for a few minutes (3–5 min) before the pH was adjusted to neutrality with dilute aqueous HCl, the mixture was extracted (ether) and dried (Na_2_SO_4_), and the solvent evaporated. The crude residue was analyzed for NMR and GC-MS. It was then purified by column chromatography and identified by the usual spectroscopic methods and comparison with authentic samples. ^1^H NMR (CDCl_3_; 300 MHz): δ 1.26 (3H, *d*, *J* = 6.5 Hz, CH_3_); 3.86 (3H,* s*, 3'-OMe); 4.37 (1H, *m*, H-2); 6.14 (1H, *dd*, *J* = 15.5 and 6.0 Hz, H-3); 6.45 (1H, *d*, *J* = 15.5 Hz, H-4); 6.76 (1H, *d*, *J* = 8.0 Hz, H-5'); 6.84 (1H, *dd*,* J* = 8.0 and 2.0 Hz, H-6'); 7.04 (1H, *d*,* J* = 2.0 Hz, H-2'); ^13^C NMR (CDCl_3_; 75 MHz): δ 23.3 (CH_3_); 56.0 (3'-OCH_3_); 68.5 (C-2); 110.1 (C-2'); 115.7 (C-5'); 120.4 (C-6'); 129.1 (C-4); 129.9 (C-1'); 132.1 (C-3); 146.7 (C-4'); 148.3 (C-3'). GC:RT = 7.47 min. EI-MS *m*/*z* 194 [M^+^].

### 4.10. Chiral-HPLC Analyses

A Varian ProStar chromatograph equipped with UV-V detector ProStar 320 at 254 nm, and a 25 cm Chiralcel OD-H column was used for analyzing the biotransformation products containing chiral **2** and synthetic *rac*-**2**. Hex and isopropanol HPLC-grade (88:12) were used as eluent. Flow rate was 0.8 mL/min, with retention times of 15.7 and 18.7 min for (*R*)- or (*S*)-enantiomer, respectively.

### 4.11. Antifungal Evaluation

#### 4.11.1. Microorganisms and Media

For the antifungal evaluation, standardized strains from the American Type Culture Collection (ATCC), Rockville, MD, USA, and Culture Collection of Reference Center of Mycology (CCC), Faculty of Biochemical and Pharmaceutical Sciences, Suipacha 531-(2000)-Rosario, Argentina were used: *C. albicans* ATCC 10231, *S. cerevisiae* ATCC 9763, *C. neoformans* ATCC 32264, *T. rubrum* CCC 110, *T. mentagrophytes* ATCC 9972 and *M. gypseum* CCC 115. Strains were grown on Sabouraud-chloramphenicol agar slants for 48 h at 30 °C, maintained on slopes of Sabouraud-dextrose agar (SDA, Oxoid, Hampshire, UK) and sub-cultured every 15 days to prevent pleomorphic transformations. Inocula of cell or spore suspensions were obtained according to reported procedures [37,38] and adjusted to 1–5 ×10^3^ cells/spores with colony forming units (CFU)/mL.

#### 4.11.2. Antifungal Susceptibility Testing

Minimum Inhibitory Concentration (MIC) of each compound was determined by using broth microdilution techniques according to the guidelines of CLSI for yeasts and for filamentous fungi [37,38]. The culture medium was RPMI-1640 (Sigma) buffered to pH 7.0 with MOPS (Remel Inc., Lenexa, KS, USA). For the assay, stock solutions of pure compounds were two-fold diluted with RPMI from 1000 to 0.98 µg/mL (final volume = 100 µL) and a final DMSO concentration ≤1%. A volume of 100 µL of inoculum suspension was added to each well with the exception of the sterility control where sterile water was added to the well instead. Terbinafine and amphotericin B were used as positive controls. Microtiter trays were incubated at 35 °C for yeasts and at 28–30 °C for the rest of fungi in a moist, dark chamber. MIC, which was defined as the lowest concentration of drug resulting in total inhibition of visual growth compared to the growth in the control wells, were visually recorded at 48 h for yeasts, and at a time according to the control fungus growth, for the rest of fungi. Compounds with MICs ≥ 1000 µg/mL were considered inactive.

## 5. Conclusions

We report here an efficient, clean, inexpensive and “natural” way to obtain the flavoring agent **1** from **3** by biotransformation with filamentous fungi and, in addition, we were able to produce (*R*)-**2** for the first time in only one bioconversion step in high conversion rates and *ee*. This work also provides a new alternative to produce (*S*)-**2** with filamentous fungi. Regarding biological activities, (*R*)- but not (*S*)-**2** showed antifungal activities against clinically important fungi, adding a new proof on the differences in biological behavior of enantiomers. These findings can also constitute an important alternative in the synthetic processes in which *rac*-**2** was used [22].
